# Comparison of Clinical and Radiographic Outcomes Following Early Anterior Cruciate Ligament Reconstruction Versus Delayed Reconstruction for Skeletally Immature Patients Using a Physeal-Sparing Anatomical Double-Bundle Reconstruction Technique

**DOI:** 10.7759/cureus.85464

**Published:** 2025-06-06

**Authors:** Kousuke Shiwaku, Tomoyuki Suzuki, Shutaro Fujimoto, Hidenori Otsubo, Takashi Matsumura, Makoto Emori, Atsushi Teramoto

**Affiliations:** 1 Orthopaedic Surgery, Sapporo Medical University School of Medicine, Sapporo, JPN; 2 Sport Medical Center, Obihiro Kyokai Hospital, Obihiro, JPN; 3 Orthopaedic Surgery, Sapporo Maruyama Seikeigeka Hospital, Sapporo, JPN; 4 Orthopaedic Surgery, Sapporo Sports Clinic, Sapporo, JPN

**Keywords:** anatomical, anterior cruciate ligament reconstruction, double-bundle, open physis, physeal-sparing, skeletally immature

## Abstract

Purpose

In anterior cruciate ligament (ACL) injury among young patients, non-operative treatments are the common approach. However, meniscal and cartilage damage have been associated with this treatment, highlighting the need to explore surgical procedures for treating pediatric ACL injuries. We employed a modification of the hybrid physeal-sparing and anatomical double-bundle ACL reconstruction (ACLR) in skeletally immature patients. Here, we aimed to compare the clinical and radiographic outcomes, including International Knee Documentation Committee (IKDC) scores and growth disturbances, between early ACLR (ER) and delayed ACLR (DR) using our modified hybrid physeal-sparing anatomical double-bundle ACL reconstruction technique in skeletally immature patients.

Methods

Patients with an ACL injury with open physes following two techniques, were retrospectively included. The patients decided whether to select ER or DR. Patients who selected ER underwent surgery as early as possible. Patients who chose DR underwent conservative treatment and anatomical double-bundle ACLR after skeletal maturation. Clinical outcomes were described using the International Knee Documentation Committee Subjective Knee Form, the Tegner activity scale, and the Lysholm score. Using bilateral standing long-leg radiographs after skeletal maturation, growth disturbance was defined as a leg length discrepancy of >10 mm or varus or valgus angular deformity of >3° compared to the other leg. For the femur, three out of 10 patients (30%) had an angle deformity of >3°. For the tibia, one in 10 patients (10%) had an angle deformity of >3°.

Results

Sixteen patients, 10 (five boys and five girls) treated with ER and six (four boys and two girls) with DR, were analyzed. Clinical outcomes of both groups were good, and there were no significant differences. None of the patients had a leg length discrepancy of >10 mm. Four of the 10 patients (40%) had angle deformity of >3°.

Conclusions

Anatomical double-bundle ACLR using a hybrid physeal-sparing technique for skeletally immature patients revealed good clinical outcomes. Even though it was not associated with any symptoms, a relatively high rate of angle deformity was observed with ER.

## Introduction

The incidence of anterior cruciate ligament (ACL) injuries has been increasing among young patients [1-4]. Recent epidemiological studies have reported an increasing incidence of ACL injuries among skeletally immature patients, approximately 50 per 100,000 in pediatric and adolescent populations [1,3]. Recovery after ACL reconstruction (ACLR) in this age group generally requires nine to 12 months before full return to sports is recommended, depending on physical recovery and psychological readiness [5, 6]. Nonoperative treatment has been the standard for pediatric patients with ACL injuries, due to concerns about physeal injury and growth disturbances that may result from surgical treatment [5-7]. However, several studies have reported secondary meniscal and cartilage damage with non-operative treatment [8,9], and early ACLR is increasing in patients with open physes [6].

Several ACLR procedures have been described [2,10-14]. An all-epiphyseal technique was reported to minimize the risk of physeal injury [11]. In this technique, both the femoral and tibial tunnels were created anatomically. However, the creation of all-epiphyseal tunnels is constrained by the size of the epiphysis and the angle necessary to keep tunnels away from the physes. Therefore, a hybrid physeal-sparing technique has been reported [14]. This technique uses an epiphyseal tunnel in the femur and a transphyseal tunnel in the tibia.

We employed a modification of the hybrid physeal-sparing technique, i.e., the hybrid physeal-sparing and anatomical double-bundle ACLR, in skeletally immature patients. This technique was intended to provide anatomical reconstruction and minimize the risk of growth disturbances. The purpose of the current study was to compare the clinical outcomes and radiographic findings of growth disturbances (angular deformity and leg length discrepancy) between early ACLR (ER) and delayed ACLR (DR) using a modified hybrid physeal-sparing anatomical double-bundle ACLR in skeletally immature patients.

## Materials and methods

Patients

This study was approved by the Institutional Review Board (IRB) of Obihiro Kyokai Hospital, Obihiro, Japan (approval number: 2015-12). A medical record search identified consecutive patients with an ACL injury with open physes at three related institutions between 2007 and 2019. This research has been approved by the authors’ affiliated institutions. Assessments of skeletal maturity were performed prior to surgery using radiographs, magnetic resonance imaging (MRI), and physical findings. Patients who satisfied the following conditions were considered skeletally mature: (1) disappearance of the high-signal band on T2-weighted MRI of the growth plate of the distal femur and proximal tibia and (2) Tanner stage 3 or higher. Patients who did not satisfy any condition were considered skeletally immature, and they were proposed for early ACLR (ER) or delayed ACLR (DR). The decision for treatment was multifactorial and patient-driven. Factors potentially influencing this decision included the presence or absence of meniscal injury, the status of the growth plates on MRI, parental preferences, the patient’s level of athletic activity, concerns regarding limitations during the growth period, and perceived risk of reinjury. All patients (n=20) were informed of the risks and benefits of both ER and DR to help them decide on their preferred option. Patients who selected ER (n=11) underwent surgery as early as possible after obtaining a full range of extension and 120° of knee flexion angles of the injured knee. The mean time from injury to surgery in the ER group was 1.7±1.2 months (range, 0-4 months). Patients with DR (n=9) underwent conservative treatment, which included immobilization with a brace and rehabilitation. In these cases, return to sports was allowed depending on the symptoms. After skeletal maturation, patients underwent anatomical double-bundle ACLR equivalent to that of adults, described by Shino et al. [13]. The clinical outcomes at the final follow-up were compared between the ER and DR groups. All surgical procedures were performed by a group of orthopedic surgeons trained under a standardized protocol at the same institution to ensure technical consistency. The mean duration from injury to surgery was 63 days in the ER group and 652 days in the DR group.

Surgical technique

**Figure 1:**
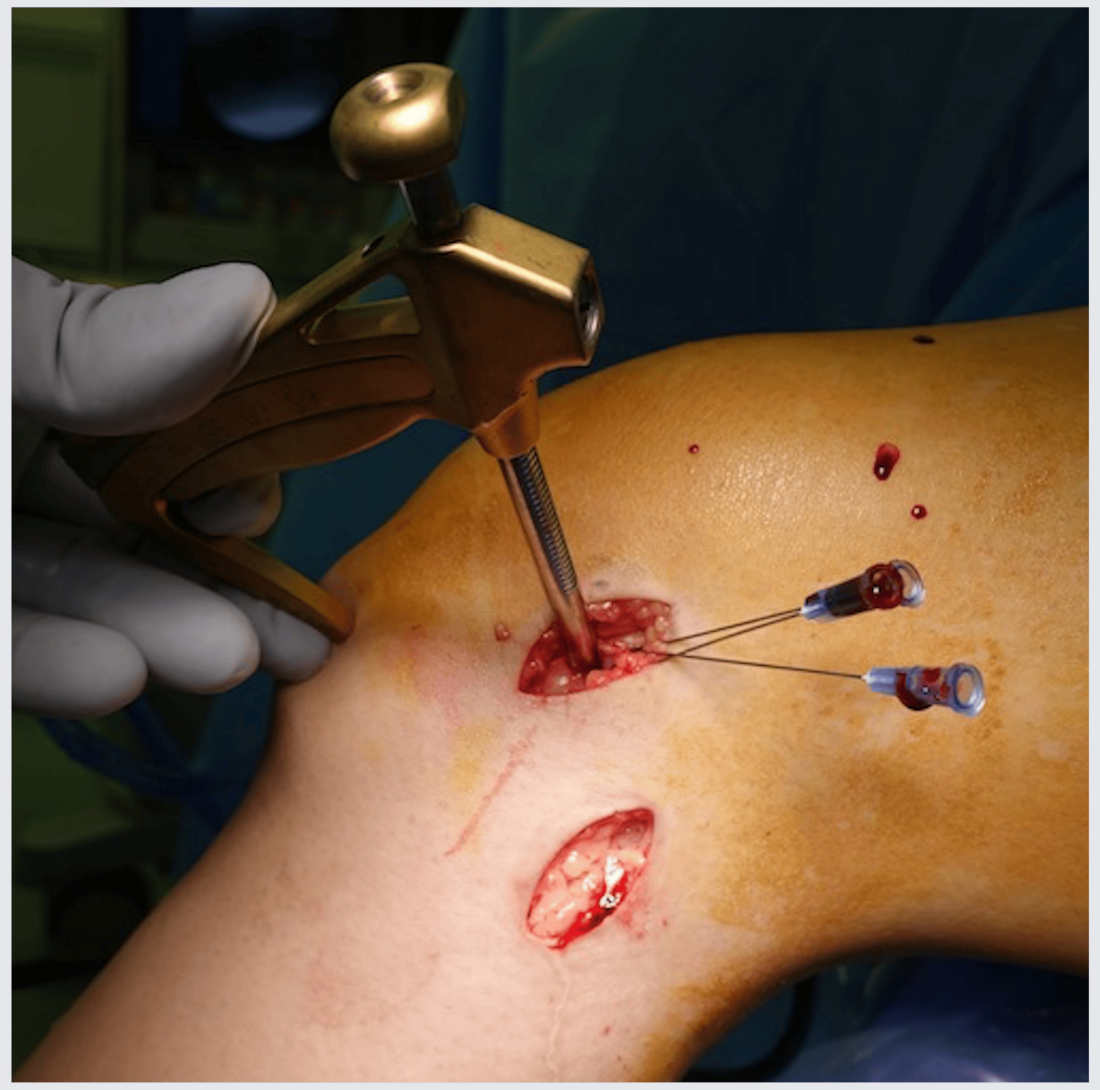
Creation of the femoral tunnel of the left knee Femoral physis is confirmed directly, and small needles are used to identify the femoral physis as needed.

**Figure 2:**
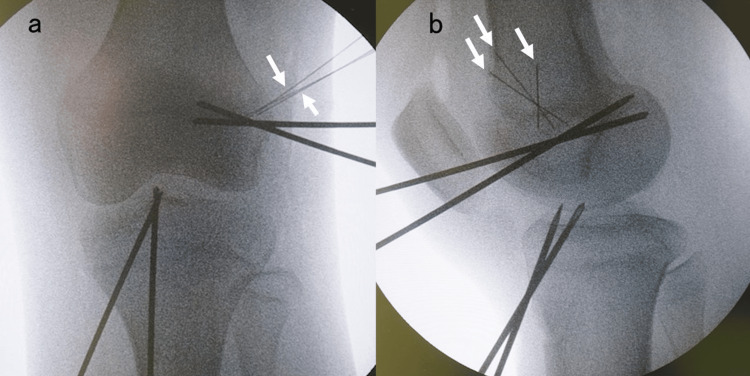
Anteroposterior (a) and lateral (b) fluoroscopic view of the left knee White arrows indicate needles whose front edges were located within the physis. It was confirmed that guide pins were not overlapping with the physis referring to the needles.

**Figure 3:**
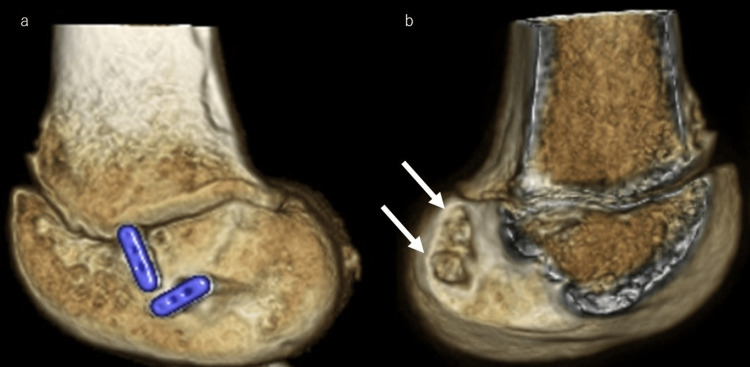
Three-dimensional CT after reconstruction of the left knee Femoral tunnels (A, B) are created within the epiphysis. White arrows indicate the locations of the femoral tunnel apertures.

Patients in the ER group were treated using a modified hybrid physeal-sparing technique with a semitendinosus tendon graft. The semitendinosus tendon was harvested using standard techniques through a 3 cm vertical skin incision. Arthroscopically, ACL remnants were debrided, and the ACL footprint was confirmed. If meniscal or chondral injury, which requires surgical treatment, was found, then the relevant treatment was performed before ACLR. A 3-4 cm skin incision was made on the lateral side of the distal femur, and the femoral physis was confirmed directly. Small needles were inserted into the femoral physis when it was visible. The posterior cruciate ligament (PCL) tibial aimer (Smith & Nephew Inc., Andover, MA, USA) was set at the footprint of the anteromedial bundle, and a guidewire was inserted into the lateral femoral epiphysis 5 mm distal to the physis (Figure 1); subsequently, a second guide pin was inserted in the same manner. Furthermore, a tibial guide (Smith & Nephew Inc.) was set at the footprint of the anteromedial bundle, and a guidewire was inserted through the anteromedial incision used for hamstring harvest. At this time, a guidewire penetrated the tibial physis. Subsequently, a second guidewire for the posterolateral bundle was inserted in the same manner. After fluoroscopic confirmation of wire placement, femoral guide wires were over-drilled to create bony tunnels (Figure 2). Tibial tunnels were created using a retro Flip Cutter (Arthrex Inc., Naples, FL, USA). Using a retro drill, sockets of depth approximately 20 mm were created for the tibial tunnels, and the diameter of penetrating tibial physis was 4 mm (Figure 3). After graft passage, fixation of the femoral side was performed using Endobutton CL (Smith & Nephew Inc.). The tibial side was fixed using a double-spike plate (DSP; Meira Corporation, Aichi, Japan) and screws. Creep of the construct was removed by repetitive manual pulls of the graft suture with the knee fixed at 15°-20°. Each graft was adjusted to 10-15 N [15].

Postoperatively, the knee was maintained at 30° of knee flexion using a brace. Range of motion was prohibited for a week after surgery. Partial weight-bearing was allowed two weeks after surgery, and full weight-bearing was allowed three weeks after surgery. Jogging was allowed 12 weeks after surgery, and return to sports was permitted after 24 weeks based on the patient's functional recovery and physician approval. This protocol was uniformly applied in both ER and DR groups.

Outcome measures

Clinical outcomes were obtained using the IKDC Subjective Knee Form [16], Tegner activity scale [17], and Lysholm score [18]. The occurrence of postoperative complications, such as ACL injury of the contralateral or ipsilateral knee and meniscal injury of the ipsilateral knee, was assessed using the medical records in both ER and DR groups. Growth disturbance was recorded as a complication of ER. Moreover, meniscal injuries that were not observed on the initial MRI were considered as injuries resulting from conservative treatment and recorded as a complication of DR.

Radiographic assessment

**Figure 4:**
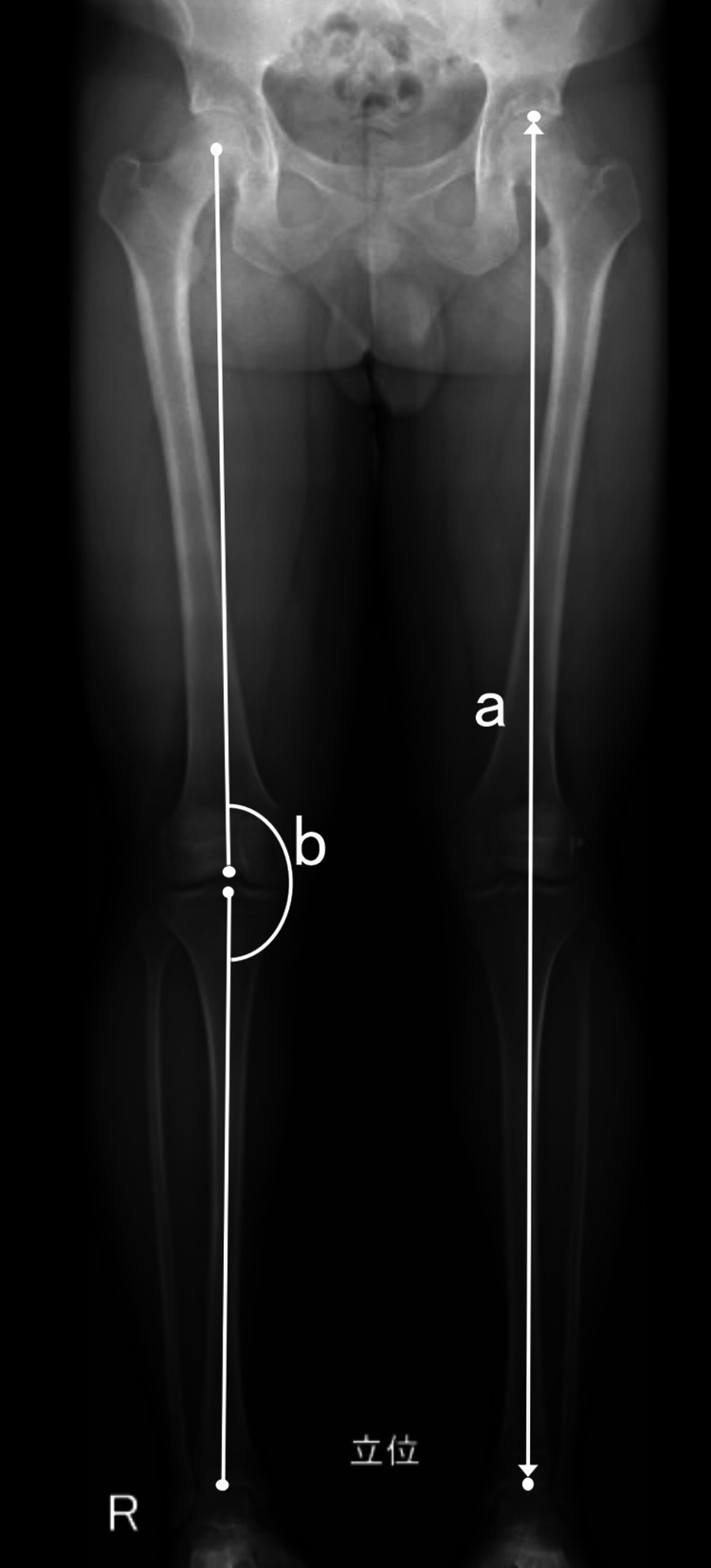
Measuring leg length and angular deformity Leg length is measured from the top of the femoral head to the center of the tibial plafond (A). Angular deformity is measured at the hip-knee-ankle angle, the angle between the femur mechanical axis and tibia mechanical axis (B).

Postoperative growth disturbances, such as leg-length discrepancies and angular deformities, were evaluated using bilateral standing long-leg radiographs after skeletal maturation. The leg length was measured from the top of the femoral head to the center of the tibial plafond. Angular deformity was measured using the hip-knee-ankle angle (HKA, the angle between the mechanical axis of the femur and the mechanical axis of the tibia, Figure 4). Growth disturbance was defined as a leg length discrepancy of >10 mm or varus or valgus angular deformity of >3°. The lateral distal femur angle (LDFA) was defined as the lateral angle between the mechanical axis and knee joint lines of the femur. The medial proximal tibial angle (MPTA) was defined as the medial angle between the mechanical axis and the knee joint lines of the proximal tibia.

Statistical analyses

Statistical analysis was performed using a t-test to compare the averages of continuous variables. A chi-squared test was used to compare the proportions of categorical variables between the two groups. The threshold for significance was set at P < 0.05. All statistical analyses were conducted using the IBM SPSS Statistics software, version 27.0 (IBM Corp., Armonk, NY, USA).

## Results

Patients

Twenty patients (10 boys and 10 girls) were identified as having ACL injury with open physis, of whom 11 patients (five boys and six girls) were treated with ER and nine patients (five boys and four girls) were treated with DR. One patient treated with ER and three patients treated with DR were lost to follow-up. Finally, 16 patients, of whom 10 (five boys and five girls) were treated with ER and 6 (four boys and two girls) were treated with DR, were analyzed in this study. The mean follow-up duration at the time of final evaluation was 1,895 days (range, X-Y days) for the ER group and 1,450 days (range, A-B days) for the DR group.

Their average age at the time of injury was 12.5±1.0 years (range, 11-14 years) for the ER group and 12.3±1.0 years (range, 11-14 years) for the DR group. The average time from injury to surgery and follow-up period was 1.7±1.2 months (range, 0-4 months) and 61.7±34.1 months (range, 22-120 months), respectively, for the ER group, and 20.8±11.4 months (range, 9-33 months) and 47.2±21.1 months (range, 27-70 months), respectively, for the DR group. The average time from injury to surgery was significantly different between the two groups (P<0.01, Table 1). No significant group differences were observed in categorical variables such as sex (df = 1, Cramer’s V = 0.408) and laterality (df = 1, Cramer’s V = 0.167).

**Table 1:** Comparison of patients' demographic data between ER and DR ER: early anterior cruciate ligament reconstruction; DR: delayed anterior cruciate ligament reconstruction.

Variables		ER	DR	P-value
		(n=10)	(n=6)	
Sex (male: female)		5:5	4:2	0.52
Laterality (right: left)		4:6	5:1	0.15
Body mass index (kg/m^2^)		20.2±2.3	22.5±5.3	0.36
Age (years)	Injury	12.5±1.0	12.3±1.0	0.75
	Surgery	12.6±0.8	14.0±2.0	0.15
Time from injury to surgery (months)		1.7±1.2	20.8±11.4	<0.01
Follow up (months)		61.7±34.1	47.2±21.1	0.37

Outcomes

The mean IKDC score was 93.8±7.8 for the ER group and 92.2±6.6 for the DR group; the mean Lysholm score was 97.7±2.2 for the ER group and 92.3±7.4 for the DR group; no significant differences (P=0.63, 0.14, respectively; Table 2) were found. The mean Tegner activity score at preinjury was 7.5±1.1 and 7.3±1.5 for the ER and DR groups, respectively, and that at final follow-up was 7.4±1.3 for the ER group and 7.3±1.5 for the DR group; no statistically significant differences (P=0.80, 0.93, respectively; Table 2) were found between the two groups. A decrease in the Tegner score was observed in only one patient who was treated with ER; all other patients maintained the same score. Three of the 10 patients in the ER group (30%) and one of the six patients (17%) in the DR group sustained ipsilateral ACL injury after returning to sports. One patient treated with ER had a partial ACL injury and did not undergo revision surgery. All other patients underwent revision surgery. One of the 10 patients in the ER group (10%) and two of the six patients in the DR group (33%) had sustained contralateral ACL injury after returning to sports. One of the 10 patients in the ER group (10%) and 2 of the 6 patients in the DR group (33%) had sustained ipsilateral meniscal injury and required meniscal repair.

**Table 2:** Comparison of clinical outcomes between ER and DR groups ER: early anterior cruciate ligament reconstruction.DR: delayed anterior cruciate ligament reconstruction; IKDC score: International Knee Documentation Committee

Variables		ER	DR	P-value
		(n=10)	(n=6)	
Lysholm score		97.7±2.2	92.3±7.4	0.14
IKDC score		93.8±7.8	92.2±6.6	0.63
Tegner Activity Scale	Pre-injury	7.5±1.1	7.3±1.5	0.80
	Follow-up	7.4±1.3	7.3±1.5	0.93

Growth disturbances were evaluated in all ER patients after skeletal maturation. None of the patients had a leg length discrepancy of >10 mm. Four of the 10 patients (40%) had an angle deformity of >3°. For LDFA, three of the 10 patients (30%) had angle deformity of >3°. For MPTA, one of the 10 patients (10%) had an angle deformity of >3°. Three patients had valgus deformities, and one patient had a varus deformity (Table 3). None of the patients with ankle deformity complained of any symptoms at the final follow-up.

**Table 3:** Radiographic assessment of growth disturbances after ER ER: early anterior cruciate ligament reconstruction; LLD: leg-length discrepancy; AD: angular deformity, positive values indicate varus, negative values indicate valgus. The numerical identifiers (e.g., 1, 2, 3, etc.) used in the table are arbitrary and were created solely for the purpose of referencing specific cases within this article. These identifiers do not correspond to any patient-identifying information.

Patients	Sex	Leg length (mm)			Hip-knee-ankle angle (°)		
		Unaffected	Affected	LLD	Unaffected	Affected	AD
1	F	794.1	787.0	7.1	180	179	1
2	F	749.9	745.4	4.5	180	181	-1
3	M	916.2	919.6	-3.4	178	182	-4
4	M	816.5	822.5	-6	180	178	2
5	M	912.0	916.9	-4.9	177	178	-1
6	M	825.7	817.0	8.7	176	183	-7
7	F	719.7	724.2	-4.5	177	177	0
8	F	759.1	757.3	1.8	180	185	-5
9	F	779.1	778.2	0.9	185	187	-2
10	M	855.5	850.6	4.9	180	176	4

Two of the six patients in the DR group had medial meniscal injuries at the time of surgery. Meniscal injury was not observed in either patient during the initial MRI.

## Discussion

The main finding of this study was that anatomical double-bundle ACLR using a hybrid physeal-sparing technique for skeletally immature patients revealed good clinical outcomes. Even though it was not associated with any symptoms, a relatively high rate of angle deformity was observed. For the femur, three of the 10 patients (30%) had an angle deformity of >3°. For tibia, one of the 10 patients (10%) had an angle deformity of >3°. Surgical treatment has been increasing as ACL injury in children has become more frequent [1].

Although there are various surgical techniques, the reports of anatomical double-bundle ACLR in skeletally immature patients are very few. This study is the first to report on the clinical outcomes and growth disturbance of anatomical double-bundle ACLR in skeletally immature patients. In this study, patients who underwent anatomical double-bundle ACLR had good clinical outcomes, and no symptomatic growth disturbance was observed.

There are several “physeal-respecting” ACLR techniques. Physeal-sparing techniques avoid the physis completely (e.g., “extraphyseal” [7, 10, 19] or “all-epiphyseal” [11-13]), while the “transphyseal” [20, 21] or “hybrid physeal-sparing” [14] techniques compromise only a small physeal area. In previous reports, clinical outcomes were generally good. The mean Lysholm score was 93-97.7, and the IKDC score or Pedi-IKDC, which is a pediatric version of the IKDC score, was 88.5-100 [7, 12-14, 19-24]. Based on previous systematic reviews and meta-analyses, the rate of leg-length discrepancies or angular deformities was 1.2%-5.8% with physeal-sparing techniques and 1.4%-1.9% with transphyseal techniques [19,22-24]. The rate of postoperative ipsilateral ACL injuries was 1.4%-7.9% with physeal-sparing techniques and 4.2%-6.2% with transphyseal techniques [19, 22-24].

In this study, ER produced satisfactory clinical outcomes similarly to DR, and the average time from injury to surgery of ER was significantly shorter than that of DR. Two of the six patients with DR had sustained meniscal injury, which was considered an injury resulting from conservative treatment; thus, ER is a good treatment method for ACL injury in skeletal immaturity contexts regarding meniscus protection. However, a relatively high rate of angle deformity should be considered after ER, although none of the patients with angle deformity complained of any symptoms at the final follow-up.

The surgical techniques used in this study were anatomical double-bundle ACLR with hybrid physeal-sparing or all-epiphyseal techniques. In the latter technique, the femoral physis was confirmed directly; therefore, iatrogenic physeal damage by drilling could be avoided more precisely than with fluoroscopy. Although creating two bone tunnels is necessary in this technique, the diameter of each tunnel was smaller than that of the single bundle reconstruction; thus, it might be easy to avoid penetrating the femoral physis. Based on the results of these ACLRs, there were some cases of angle deformities as growth disturbances, although they did not present any clinical symptoms. No leg-length discrepancy of >10 mm was observed. In previous reports, <10° to 15° angle deformity and <15 mm leg-length discrepancy were not associated with knee arthritis [25, 26]. Considering that three of the 10 patients (30%) had angle deformity of >3° for the femur and one of the 10 patients (10%) had angle deformity of >3° for the tibia, other optional surgical procedures, including over-the-top, may be considered in the future.

In previous studies in adults, the postoperative anterior and rotational stability after double-bundle ACLR was significantly better than that after single-bundle reconstruction, although there were no significant differences in clinical outcomes [27, 28]. Although clinical outcomes after single-bundle reconstruction in skeletally immature patients were good, anatomical double-bundle reconstruction is one of the considerable choices to obtain good knee stability equivalent to reconstruction for adults, even for skeletally immature patients.

In this study, the incidence rates of postoperative ipsilateral and contralateral ACL injuries were 30% and 10%, respectively. These values are higher than those reported in previous studies; the reason for this difference was speculated as follows: (1) All patients in this study returned to sport after surgery, and 90% of them returned to the same level of sport as in pre-injury, and (2) there is a possibility that the load of the graft was more at the femoral tunnel aperture because the graft bending angle of the femoral tunnel was acute due to avoidance of femoral physis. The incidence of postoperative ipsilateral and contralateral ACL injury is more frequent in younger patients; however, the reason for this is not completely understood [29, 30]. Further research is required to determine the reason for the high incidence of postoperative ACL injury with our surgical technique.

In the present study, the ipsilateral reinjury rate in the ER group reached 30%, which appears higher than previously reported. This may be attributed to two main factors: (1) all patients returned to sports after surgery, and 90% resumed participation at the same level as before injury, and (2) to avoid violating the femoral physis, the graft bending angle at the femoral tunnel aperture was more acute, possibly increasing mechanical stress at the graft interface. Additionally, the biological vulnerability of skeletally immature patients may have contributed to the elevated reinjury rate through a multifactorial mechanism.

While the findings suggest that early ACL reconstruction may offer benefits such as preservation of meniscal integrity and timely functional recovery, these conclusions should be interpreted with caution. The small sample size and patient-driven treatment selection inherently limit generalizability. The influence of unmeasured confounding variables-such as motivation to return to sports or parental decision-making-cannot be excluded. Further prospective, multi-institutional studies are warranted to validate these preliminary results.

Although the angular deformities observed in the ER group were asymptomatic at final follow-up, even mild alterations in lower limb alignment during skeletal development may affect long-term joint loading patterns and increase the risk of cartilage degeneration. From a biomechanical standpoint, angular deviations of just a few degrees can lead to altered stress distribution and thus should not be dismissed. Careful long-term surveillance remains essential.

This study had several limitations. First, the number of patients in this study was small. Second, the treatment decision was multifactorial and patient-driven, which may have led to selection bias. Third, the follow-up duration was not enough and varied among patients, which may have affected the comparability of long-term outcomes between the ER and DR groups. Fourth, the lack of biomechanical evaluation of knee function remains a limitation of this study. This aspect should be addressed in future investigations. Fifth, we have acknowledged that the treatment decision was influenced by multiple subjective and clinical factors, including meniscal pathology, skeletal maturity, parental input, and athletic goals, which may have introduced unmeasured confounders and affected the comparability between groups.

## Conclusions

Anatomical double-bundle ACLR using a hybrid physeal-sparing technique for skeletally immature patients revealed good clinical outcomes. The anatomical double-bundle reconstruction is a considerable choice, even in skeletally immature patients. Although none of the patients with angle deformity complained of any symptoms at the final follow-up, the relatively high rate of angle deformity should be considered.
